# A Hypericin Delivery System Based on Polydopamine Coated Cerium Oxide Nanorods for Targeted Photodynamic Therapy

**DOI:** 10.3390/polym11061025

**Published:** 2019-06-10

**Authors:** Yang Wang, Yu Zhang, Ming Jin, Yinghua Lv, Zhichao Pei, Yuxin Pei

**Affiliations:** Shannxi Key Laboratory of Natural Products & Chemical Biology, College of Chemistry & Pharmacy, Northwest A&F University, Yangling 712100, China; ywang9601@nwafu.edu.cn (Y.W.); zyu807158119@163.com (Y.Z.); jinming@nwafu.edu.cn (M.J.); hongriyinghua@163.com (Y.L.); peizc@nwafu.edu.cn (Z.P.)

**Keywords:** cerium oxide nanorods, hypericin, targeted delivery, photodynamic therapy, polydopamine

## Abstract

Photodynamic therapy (PDT) as a non-aggressive therapy with fewer side effects has unique advantages over traditional treatments. However, PDT still has certain limitations in clinical applications, mainly because most photosensitizers utilized in PDT are hydrophobic compounds, which will self-aggregate in the aqueous phase and cause undesirable effects. In order to resolve this, we utilized the self-polymerization of dopamine molecules under alkaline conditions to coat cerium oxide nanorods (CeONR) with a dense polydopamine (PDA) film. Thereafter, thiolated galactose (Gal-SH) and hypericin (Hyp) were modified and loaded onto the surface to construct CeONR@PDA-Gal/Hyp, respectively, which can be used for targeted photodynamic therapy of human hepatoma HepG2 cells. CeONR@PDA-Gal/Hyp was characterized by transmission electron microscope (TEM), Zeta potential, Ultraviolet-visible (UV-Vis), and fluorescence spectroscopy, respectively. This hypericin delivery system possesses good biocompatibility and specific targeting ability, where the galactose units on the surface of CeONR@PDA-Gal/Hyp can specifically recognize the asialo-glycoprotein receptors (ASGP-R), which overexpress on HepG2 cell membrane. Furthermore, Hyp will detach from the surface of CeONR@PDA-Gal/Hyp after the nanorods enter cancer cells, and shows excellent PDT effect under the irradiation of light with a wavelength of 590 nm. Our work exemplifies a novel targeted delivery of hydrophobic photosensitizers for cancer treatment.

## 1. Introduction

Cancer is one of the major causes leading to death worldwide [1,2]. Traditional treatments, such as surgery, chemotherapy, and radiotherapy do not have ideal therapeutic effects due to various side effects [3,4]. Currently, selective killing of tumor cells and minimizing damage to normal cells is one of the biggest challenges facing oncology. Compared with the traditional treatments, photodynamic therapy (PDT) as a non-aggressive therapy has universal applicability in clinical treatment to target organs and tissues with strong killing ability and less side effects [5,6]. Today, PDT is not only used to treat cardiovascular diseases, skin diseases, eye diseases, and infectious diseases, but also to treat tumors [7,8,9].

Hypericin (Hyp) has been recognized as a potential excellent photosensitizer due to its unique photochemical property [10]. For example, Hyp has a high singlet oxygen yield (0.17–0.80) and a maximum absorption wavelength (590 nm), which is close to an optimal wavelength range (600 to 1000 nm) [11]. In addition, Hyp also has the characteristics of low cost and low dark toxicity. However, Hyp is a hydrophobic compound and forms aggregates easily in physiological conditions, which leads to its fluorescence being quenched, further greatly reduces its photodynamic effect [12,13]. This property severely limits Hyp’s clinical application. Previously, our group designed a strategy to deliver Hyp by using polydopamine coated magnetic iron oxide nanoparticles with a lactose derivative as a targeting ligand, which exhibited excellent stability and dispersibility in aqueous medium [14]. The nanoparticles protect the photosensitizer from premature release, enhance the dispersity of the photosensitizer in water, and prolonged the circulation time in blood. At the same time, the cellular-uptake of the photosensitizer is enhanced, which allows the photosensitizer to accumulate more in the lesion area [15,16,17]. Cerium oxide nanoparticles (CeONPs) attract the interest of researchers in nano-drug delivery systems and have gained rapid progress in the fields of bioanalysis and biomedicine [18,19,20,21,22]. It has been reported that CeONPs can cause cytotoxicity to tumor cells through lipid peroxidation and cell membrane leakage, but cannot cause cytotoxicity to normal cells [23,24,25]. Moreover, reactive oxygen species (ROS) can be generated when Ce (III) is oxidized to Ce (IV) under an acidic environment, which leads to a synergistic therapeutic effect [26]. Therefore, CeONPs have a potential prospect in anti-tumor therapy [18,27], and the study of CeONPs as a drug carrier is of significance for enrichment of the material candidates in constructing biocompatible delivery systems.

In this work, polydopamine (PDA), as a widely applied biomimetic material with unique properties such as the universal adhesion property, excellent biocompatibility, and biodegradability [28], was coated on the surface of cerium oxide nanorods (CeONR) in situ via self-polymerization of dopamine, which acted as a basic skeleton. Thereafter, the catechol functional group on the surface of CeONR@PDA can be oxidized to quinone, which can easily undergo Michael addition reaction and Schiff base reaction with sulfhydryl and amino compounds [29]. Thiolated galactose, as a targeted unit of human hepatoma HepG2 cells, was further modified through the Michael addition reaction to construct a new type of photosensitizer delivery system (CeONR@PDA-Gal), which could be used to load Hyp onto its surface. We expect this novel platform will not only overcome the problems resulting from the hydrophobicity of Hyp, but also show excellent biocompatibility and photodynamic effect.

## 2. Materials and Methods 

### 2.1. Materials

Emodin was purchased from Xi’an Natural Field Bio-Technique Co., Ltd. (Xi’an, China). Boron trifluoride ether, dopamine hydrochloride, 2′,7′-dichlorodihydrofluorescein diacetate (DCFH-DA), sodium methoxide, and tin (II) chloride dihydrate (SnCl_2_·2H_2_O) were purchased from Shanghai Aladdin Biochemical Technology Co., Ltd. (Shanghai, China). Acetic anhydride, dimethyl formamide, and glacial acetic acid were purchased from Chengdu Chron Chemicals Co., Ltd. (Chengdu, China). Iodine, ferrous sulfate, and acetone were obtained from Tianjin Bodi Chemicals Co., Ltd. (Tianjin, China). Chromium trioxide was obtained from Sinopharm Pharmaceutical Co., Ltd. (Shanghai, China). Potassium thioacetate was purchased from Alfa Aesar (Shanghai, China). Pyridine nitrogen oxide was purchased from Sigma-Aldrich (Shanghai, China). 2-chloroethoxyl-2-ethoxydiethanol was purchased from Tianjin Xiensi Biochemical Technology Co., Ltd. (Tianjin, China). Annexin V-FITC and propidium iodide (PI) were obtained from Yeasen Biotech Co., Ltd. (Shanghai, China). Hyp was synthesized via the method developed by our lab (Scheme S1) [30]. The synthesis method of thiolated galactose was shown in Scheme S2. The human breast adenocarci noma cells (MCF-7), human hepatoma cells (HepG2), and human embryonic kidney T cells (293T) were obtained from KeyGEM BioTECH Co., Ltd. (Nanjing, China). β-Actin (#4970), Caspase-3 (#96620), cleaved Caspase-3 (#9664), cleaved Caspase-9 (#9509), and Caspase-9 antibody (#9504) were obtained from Cell Signaling Co., Ltd. (Shanghai, China).

### 2.2. Instrumentation

Nuclear magnetic resonance (NMR) spectra were recorded by using a Bruker 500 MHz Spectrometer (Karlsruhe, Germany) in DMSO-*d*_6_ or CDCl_3_. Transmission electron microscope (TEM) analyses were done by using a Hitachi HT7700 instrument (Tokyo, Japan, 80 kV). The ultraviolet-visible (UV-Vis) spectra and fluorescence spectra were measured with the Shimadzu 1750 UV-Vis spectrophotometer (Kyoto, Japan) and Shimadzu RF-5301 fluorescence spectrometer (Kyoto, Japan) at room temperature, respectively. The Zeta potentials of the nanorods at each fabrication step were recorded by a DelsaTM Nano system (Beckman Coulter, Brea, CA, USA). The cell images were acquired with a confocal laser scanning microscope (CLSM) (Andor REVOLUTIONWD, Oxford, UK). The 590-nm and 630-nm laser instruments have a power density of 8.6 mW/cm^2^ (Shenzhen, China). Cell viability was analyzed by methyl thiazolyl tetrazolium (MTT) assay using a microplate reader (Tecan Infinite M1000 Pro, Männedorf, Switzerland).

### 2.3. Synthesis of CeONR@PDA-Gal/Hyp

#### 2.3.1. Synthesis of CeONR@PDA

Cerium oxide nanorods (CeONR) were prepared by a method that was reported previously [31]. First, CeONR (5 mg) was mixed into tris(hydroxymethyl)aminomethane hydrochloride (Tris-HCl) buffer (pH 8.5, 10 mM, 10 mL) and sonicated for 60 min to uniformly disperse the nanorods. Dopamine hydrochloride (20 mg) was added to the system under agitation and stirred for 30 min at ambient temperature. After the reaction was completed, the mixture was centrifuged for 3 min, the supernatant was removed, and the precipitate was collected. The polydopamine-coated cerium oxide nanorods (CeONR@PDA) were obtained by washing the precipitate with ultrapure water several times to remove unreacted dopamine molecules and dopamine oligomers.

#### 2.3.2. Synthesis of CeONR@PDA-Gal

The prepared CeONR@PDA was mixed into Tris-HCl buffer (pH 8.5, 10 mM, and 5 mL) and ultrasonicated for 60 min to uniformly disperse the nanorods. Furthermore, thiolated galactose (Gal-SH) (20 mg) was added to the reaction mixture, and then the mixture was stirred for 6 h at ambient temperature. The mixture was centrifuged for 3 min after the reaction completed. Then the supernatant was removed and the precipitate was collected. The galactose-modified drug carrier (CeONR@PDA-Gal) was obtained by washing the precipitate with ultrapure water several times to remove the unreacted Gal-SH.

#### 2.3.3. Synthesis and loading capacity of CeONR@PDA-Gal/Hyp

The prepared CeONR@PDA-Gal was added to methanol (4 mL) and ultrasonicated for 60 min to uniformly disperse the nanorods. Hyp (2 mg) was added to the system and stirred for 24 h at ambient temperature in the dark. Hyp loaded onto the surface of the nanorods through π–π stacking interactions and hydrogen bonding. After completion of the reaction, the mixture was centrifuged for 3 min. The precipitate was collected and washed with methanol several times to remove the unreacted Hyp. The supernatant was combined, and the content of Hyp in the supernatant was measured by UV-Vis spectrophotometry. The loading capacity of Hyp (C_H_) was calculated by using Formula (1).

(1) $$C_{H}\left(\%\right)=\frac{M_{F}-M_{S}}{M_{C}+{\;M}_{F}-M_{S}}\times 100\%$$

where M_F_ is the feeding amount of free Hyp, M_S_ is the amount of free Hyp in the collected supernatant, and M_c_ is the feeding amount of CeONR. The loading capacity of Hyp is 19.96%.

### 2.4. Release Profiles of Hyp

CeONR@PDA-Gal/Hyp (15 mg) was dispersed in phosphate buffer saline (PBS) (3 mL, pH 7.4) and transferred into three dialysis bags (molecular weight cutoff = 8000, respectively) evenly. Then the bags were submerged at 4 mL PBS with different pH values (7.4, 5.0, and 4.0) and stirred. At specified time intervals, 0.1 mL of the release medium was taken out for measuring the release of Hyp with a microplate reader, and then was returned to the original release medium.

### 2.5. Characterization of CeONR@PDA-Gal/Hyp

The prepared CeONR and CeONR@PDA-Gal/Hyp were dispersed into PBS (pH 7.5, 10 mM) via ultrasonication. The suspension was dropped onto a copper mesh. The size and morphology of the nanorods were observed by a TEM. The Zeta potential of the nanorods was measured by using a dynamic light scattering (DLS) instrument. The UV-Vis absorption spectra were recorded using a Shimadzu UV-Vis spectrophotometer. The fluorescence spectra were recorded using a Shimadzu fluorescence spectrometer. The Fourier transform infrared (FT-IR) spectra were taken using an FT-IR instrument.

### 2.6. Cellular-Uptake Experiment

HepG2 cells were seeded and cultured for 24 h in 35-mm plastic bottomed μ-dishes. The culture medium was replaced with new medium containing CeONR@PDA-Gal/Hyp (Hyp equivalent of 1 μM). The cells were further cultured for 2, 4, or 12 h, respectively. Then, HepG2 cells were washed with PBS (pH 7.4, 2 mL × 3) to remove the nanorods which were not taken by the cells. Subsequently, the cells were treated with formaldehyde (4.0%, 0.5 mL) for 10 min. Then the fixed cells were washed by using PBS (pH 7.4, 2 mL) three times. Hoechst 33258 (0.5 mL) was added and left to stain for 5 min. After washing three times by PBS (pH 7.4, 2 mL), the fluorescence of Hoechst 33258 and Hyp were studied by CLSM. In addition, the fluorescence intensity of Hyp entering the HepG2 cells was analyzed by using Image-Pro Plus software. The Hoechst 33258 was detected at a blue channel (447 ± 30 nm) with an excitation of 405 nm. Hyp was detected at a red channel (607 ± 18 nm) with an excitation of 561 nm.

### 2.7. Targeting Ability Experiment

HepG2 cells and MCF-7 cells were separately seeded and cultured for 24 h in 35-mm plastic bottomed μ-dishes. Furthermore, the previous culture medium was removed and new culture medium containing CeONR@PDA-Gal/Hyp (Hyp equivalent of 1 μM) was added. In contrast, another group of HepG2 cells was pre-incubated for 4 h with lactobionic acid (LBA, 2 mg/mL, 1 mL). LBA can occupy the galactose receptors on the cytomembrane and inhibit the targeting of the nanorods. After washing with PBS (pH 7.4, 2 mL) three times, a fresh medium containing CeONR@PDA-Gal/Hyp (Hyp equiv of 1 μM) was added. Both groups were cultured for 4 h. The cells were washed by using PBS (pH 7.4, 2 mL) three times to remove the non-uptaken nanorods. Subsequently, the cells were fixed with formaldehyde (4.0%, 0.5 mL) for 10 min. The fixed cells were then washed by using PBS (pH 7.4, 2 mL) three times. Hoechst 33258 (0.5 mL) was then added and left to stain for 5 min. Then the fluorescence of Hoechst 33258 and Hyp were captured by different channels of the confocal laser, after washing with PBS (pH 7.4, 2 mL) three times. The fluorescence density of Hyp entering the cells was analyzed using Image-Pro Plus software. The Hoechst 33258 was detected at a blue channel (447 ± 30 nm) with an excitation of 405 nm. Hyp was detected at a red channel (607 ± 18 nm) with an excitation of 561 nm.

### 2.8. Intracellular ROS Levels

The generation of intracellular ROS was measured through the 2′,7′-dichlorodihydrofluorescein diacetate (DCFH-DA) method. MCF-7 and HepG2 cells were seeded and cultured for 24 h in six-well plates covered with coverslips. The previous culture medium was removed and fresh medium that contains PBS, Hyp (1 μM), or CeONR@PDA-Gal/Hyp (Hyp equivalent of 1 μM) was added, respectively. After co-cultivation for 4 h, the previous culture medium was removed and the cells were washed by using PBS (pH 7.4, 2 mL) three times to remove the non-uptake nanorods. Furthermore, DCFH-DA solution (20 μM, 1 mL) was added to the six-well plate, cultured for 30 min, and washed by using PBS (pH 7.4, 2 mL) three times. The HepG2 and MCF-7 cells were immediately irradiated for 30 min by a 590-nm laser, which has a power density of 8.6 mW/cm^2^. The fluorescence intensity of DCF was observed with a fluorescence microscope at a green channel (525 ± 25 nm) with an excitation of 488 nm.

### 2.9. Phototoxicity 

HepG2 cells and 293T cells were seeded and cultured for 24 h in 96-well plates, respectively. The previous culture medium was removed, and fresh medium, which contains different concentrations of Hyp and CeONR@PDA-Gal/Hyp, was added, respectively. The concentrations relative to Hyp were 0.025, 0.05, 0.075, 0.1, 0.125, 0.15, 0.25, 0.3, 0.4, or 0.5 μM, respectively. The medium was removed after co-cultivation of the drugs with the cells for 4 h. Then, the HepG2 cells and 293T cells were washed by using PBS (pH 7.4, 2 mL) three times to remove the non-uptaken nanorods. The cells were immediately irradiated for 30 min with a light of 590 nm. The cells were cultured for another 24 h under the dark. As a control, the same concentration gradient of CeONR@PDA-Gal/Hyp was co-cultured with HepG2 cells, but the cells were not exposed to light. MTT assay was used to determine the cell viabilities.

### 2.10. Material Toxicity

HepG2 cells and 293T cells were seeded and cultured for 24 h in 96-well plates. The previous culture medium was removed, and fresh medium, which contains different concentrations of CeONR@PDA-Gal, was added. The cells were then co-cultured in the dark for 24 h. The concentrations relative to cerium oxide were 5, 15, 20, 25, or 50 μM, respectively. MTT assay was used to determine the cell viabilities.

### 2.11. Material Biocompatibility

The 293T cells were seeded and cultured for 24 h in 96-well plates. The previous culture medium was removed, and fresh medium, which contains different concentrations of Hyp, CeONR@PDA-Gal, and CeONR@PDA-Gal/Hyp, was added and then co-cultured in the dark for 24 h. The concentrations relative to Hyp were 0.05, 0.3, 0.4, 0.5, or 1 μM, respectively. The MTT assay determined the cell viabilities.

In addition, 0.25 μM Hyp and CeONR@PDA-Gal/Hyp were added to 96-well plates inoculated with 293T cells, respectively. Under dark conditions, they were co-cultured for 24, 48, or 72 h, respectively. MTT assay was used to determine the cell viabilities.

### 2.12. Cell Apoptosis Experiments

#### 2.12.1. Hoechst 33258 Staining Assay

HepG2 cells were seeded and cultured for 24 h in a 35-mm plastic bottomed μ-dishes. The previous culture medium was removed and fresh medium containing PBS or CeONR@PDA-Gal/Hyp (Hyp equivalent of 1 μM) was added, separately. After incubated for 4 h, HepG2 cells were washed by using PBS (pH 7.4, 2 mL) three times to remove the non-uptaken nanorods. The cells were immediately irradiated for 30 min by a 590-nm laser, and then incubated for 24 h in the dark. Subsequently, the previous medium was removed and the cells were fixed with formaldehyde (4.0%, 0.5 mL) for 10 min. The fixed cells were then washed by using PBS (pH 7.4, 2 mL) three times. Hoechst 33258 (0.5 mL) staining solution was mixed with the cells and left to stain for 5 min. After washed with PBS (pH 7.4, 2 mL) three times, the fragmented nuclei and condensed chromatin in Hoechst 33258-stained apoptotic cells were viewed by CLSM. Hoechst 33258 was detected at a blue channel (447 ± 30 nm) with an excitation of 405 nm.

#### 2.12.2. Annexin-V-FITC/PI Staining Assay

HepG2 cells were seeded and cultured for 24 h in six-well plates. The previous culture medium was removed and fresh medium containing PBS or CeONR@PDA-Gal/Hyp (Hyp equiv of 1 μM) was added, respectively. Subsequently, HepG2 cells were incubated for 4 h and washed with PBS (pH 7.4, 2 mL) three times to remove the non-uptaken nanorods. The cells were immediately irradiated for 30 min by a 590-nm laser, and then incubated for 24 h in the dark. Thereafter, the cells were harvested, washed by using PBS (pH 7.4, 2 mL) three times, and resuspended in 500 μL PBS (pH 7.4). Annexin V-FITC (5 μL) and PI (10 μL) were added into the cells. The cells were incubated for 15 min in the dark at ambient temperature and analyzed by flow cytometry.

#### 2.12.3. Western Blot Analysis 

HepG2 cells were seeded and cultured for 24 h in six-well plates. The previous culture medium was removed and fresh medium containing PBS or CeONR@PDA-Gal/Hyp (Hyp equivalent of 1 μM) was added, respectively. After incubated for 4 h, HepG2 cells were washed using PBS (pH 7.4, 2 mL) three times to remove the non-uptaken nanorods. One of the two groups co-cultured with CeONR@PDA-Gal/Hyp was subjected to light treatment, and the other was not. The cells were lysed in RIPA buffer supplemented with protease and phosphatase inhibitors on ice for 20 min. The lysates were stored in ice water and proceeded for ultra-sonication (60 s/burst at 30 W for 30 times). The debris was separated from the soluble fraction by centrifugation at 12,000 g for 15 min at 4 °C. The lysates were mixed with 6 × SDS loading buffer and boiled at 100 °C for 10 min. The lysates containing equal amount of protein (20 μg) were analyzed by SDS-PAGE, which was followed by Western blot, according to the standard procedure.

## 3. Results and Discussion

### 3.1. Synthesis and Characterization of CeONR@PDA-Gal/Hyp

**Scheme 1:**
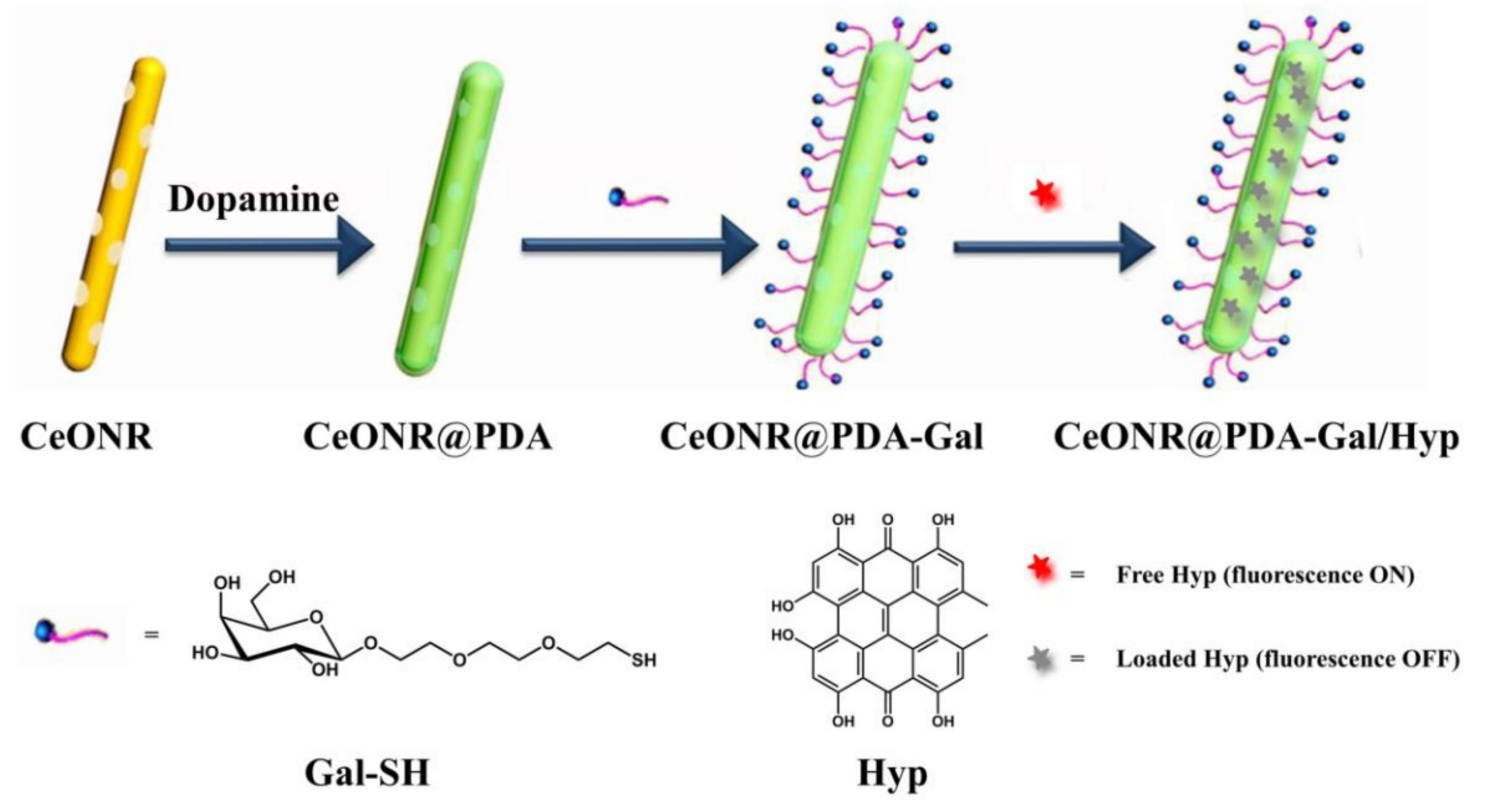
Illustration of the construction process of CeONR@PDA-Gal/Hyp.

**Figure 1:**
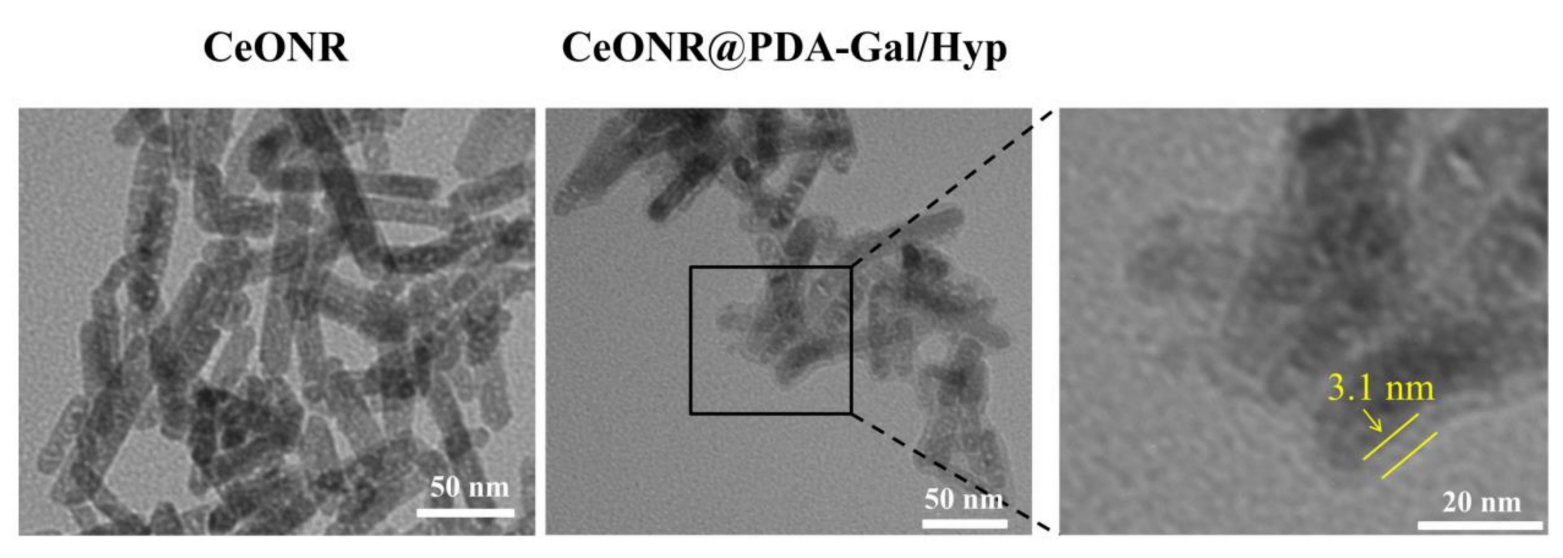
TEM images of CeONR and CeONR@PDA-Gal/Hyp nanorods (Scale bar: 50 nm for the right and middle images as well as 20 nm for the left image).

The construction process of CeONR@PDA-Gal/Hyp is illustrated in Scheme 1. First, a dense PDA film was formed on the surface of CeONR through the self-polymerization of dopamine under oxygen and alkaline conditions (pH = 8) [32,33] to obtain CeONR@PDA. PDA film not only endows the system with good biocompatibility and biodegradability under physiological conditions, but also facilitates the secondary modification and provides the possibility of modifying the target molecules to the surface of the nanorods. Thiolated galactose (Gal-SH) was modified onto the surface of CeONR@PDA as a targeting molecule for HepG2 cells. Thereafter, Hyp can be loaded onto the surface of CeONR@PDA-Gal by π–π stacking and hydrogen bonding to overcome the problem of poor solubility of Hyp in an aqueous phase [34,35]. The size and morphology of CeONR and CeONR@PDA-Gal/Hyp were revealed by TEM (Figure 1). A layer with a thickness of 3.1 nm was observed on the surface of CeONR@PDA-Gal/Hyp, which indicated that PDA was successfully coated on the surface of CeONR. The average length and diameter of CeONR@PDA-Gal/Hyp, 61 ± 0.9 nm and 12.5 ± 1.2 nm, respectively, were statistically obtained using Image-Pro Plus software (n = 100).

**Figure 2:**
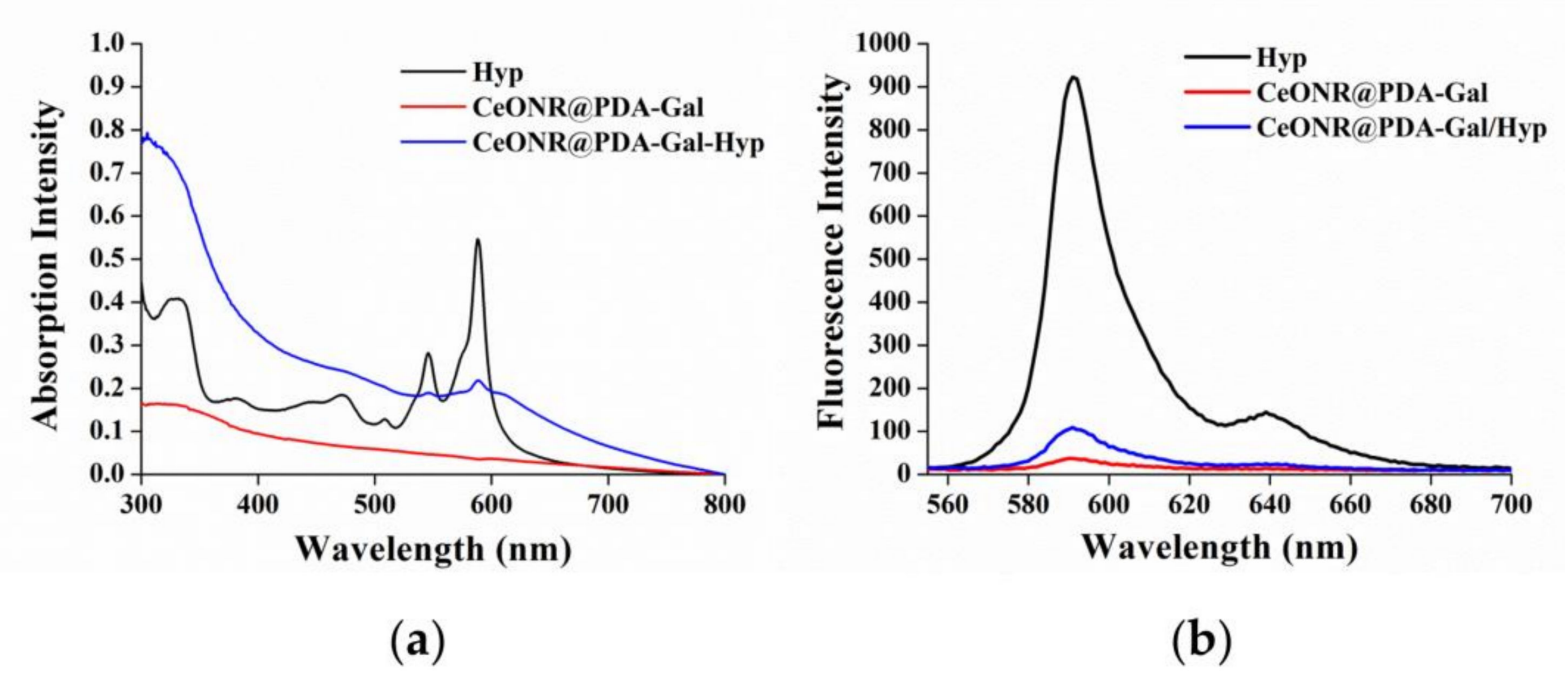
(**a**) UV-Vis spectra and (**b**) fluorescence spectra of Hyp, CeONR@PDA-Gal, and CeONR@PDA-Gal/Hyp in methanol. The amount of Hyp in CeONR@PDA-Gal/Hyp was the same as that in the methanol solution of Hyp.

UV-Vis spectroscopy and fluorescence spectroscopy were used to demonstrate that Hyp had been successfully loaded onto the CeONR@PDA-Gal surface. UV-Vis spectra revealed that CeONR@PDA-Gal/Hyp (blue line) had two characteristic absorption peaks of Hyp at 590 nm and 546 nm compared to CeONR@PDA-Gal (red line), which demonstrated that Hyp was successfully loaded onto the surface of the CeONR@PDA-Gal nanorods (Figure 2a). It has been reported that, when a photosensitizer is loaded onto the surface of PDA by stacking and hydrogen bonding, its fluorescence would be quenched by PDA [36,37]. The successful loading of Hyp was fully demonstrated by fluorescence spectroscopy. The fluorescence intensity of CeONR@PDA-Gal/Hyp (blue line) at 590 nm was significantly weaker than that of Hyp (black line) at the same concentration, which further demonstrated the successful loading of Hyp and quenched fluorescence of Hyp as expected (Figure 2b). The presence of PDA film and Gal-SH on the surface of CeONR was verified by the characteristic absorption peaks at 1290 cm^−1^ (C−N−C stretching vibration) and 2935 cm^−1^ (C-H stretching vibration) in the FT-IR spectrum of CeONR@PDA-Gal/Hyp, respectively (Figure S1).

As listed in Table 1, the negative surface charge (−8.46 ± 0.7 mV) of CeONR in water was indicated by Zeta potential results, and then, with the modification of the CeONR, the charge of the nanorods became more negative. It could, therefore, be shown from the Zeta potential data that the modification of the nanorods at each step was carried out smoothly.

### 3.2. Cellular Uptake

**Figure 3:**
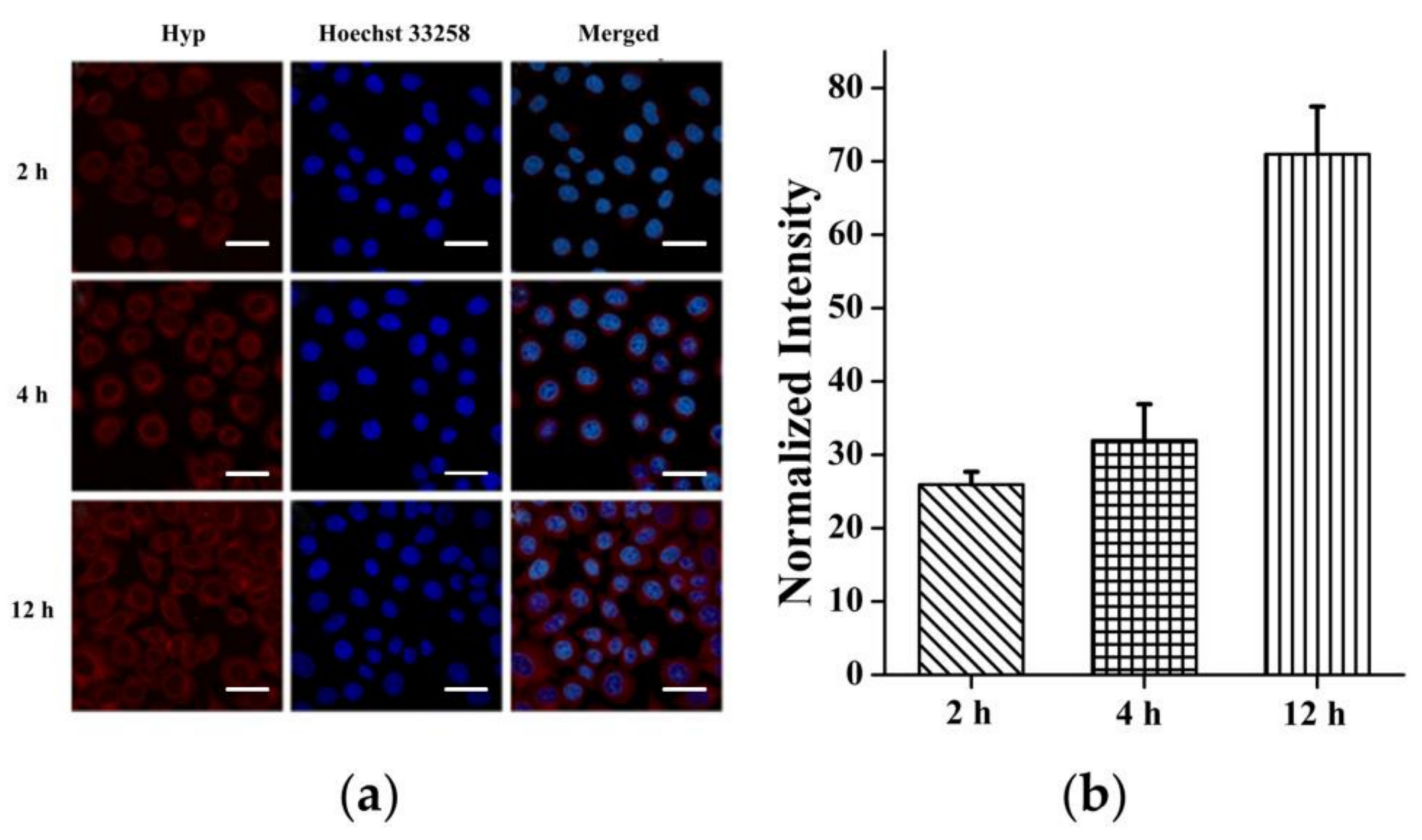
(**a**) CLSM images of HepG2 cells after various periods of incubation with CeONR@PDA-Gal/Hyp (2, 4, and 12 h), respectively. Scale bar: 20 μm. (**b**) Quantitative analysis of the fluorescence of Hyp in HepG2 cells by Image-Pro Plus (n = 50).

To assess the uptake capacity of HepG2 cells to CeONR@PDA-Gal/Hyp, CeONR@PDA-Gal/Hyp (0.25 μM Hyp, 0.5 μg/mL CeONR) was co-cultured with HepG2 cells for 2, 4, and 12 h, respectively. Initially, the fluorescence of Hyp on CeONR@PDA-Gal/Hyp was quenched by PDA [36]. Once Hyp is released, the fluorescence of Hyp will be restored. Therefore, CLSM was used to observe the change of Hyp fluorescence with incubation time, which further indirectly revealed the release of Hyp. The fluorescence of Hyp increased with the prolongation of incubation time (Figure 3a), which indicates CeONR@PDA-Gal/Hyp was efficiently taken up by HepG2 cells and Hyp was released slowly after the nanorods entered the cells. Considering the release rate of Hyp at a low pH is relatively low (Figure S2), we speculate that there may be some other unknown stimuli that contributes to the release of Hyp under the intracellular environment. Furthermore, the CLSM data were semi-quantitatively analyzed by Image-Pro Plus software (Figure 3b). The trend of fluorescence changes in Figure 3a matched well with the calculated results, and the histogram distinctly showed the amount of Hyp entering the cells that increase with time, from 2 h to 12 h.

### 3.3. Targetability of CeONR@PDA-Gal/Hyp

**Figure 4:**
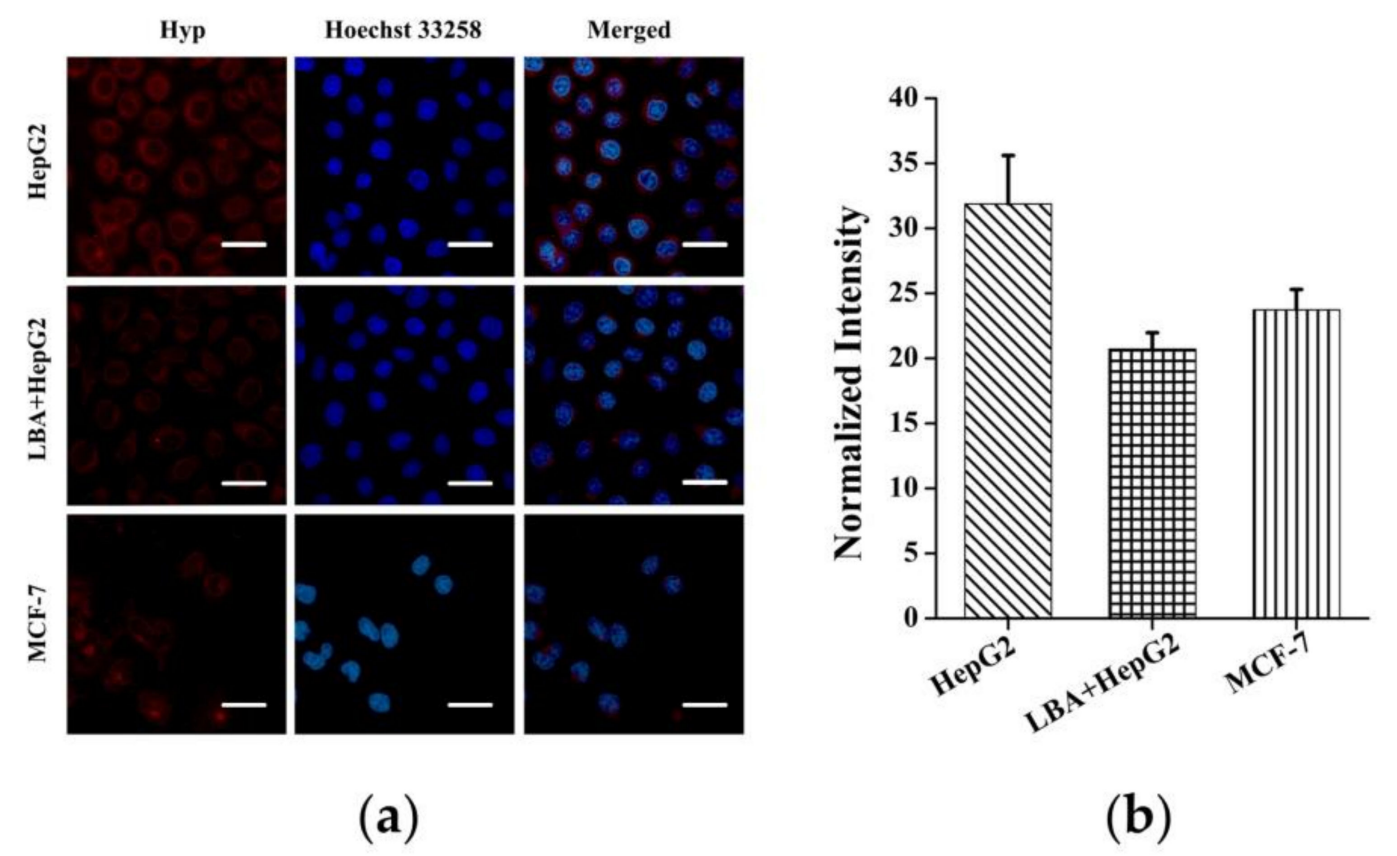
(**a**) CLSM images of HepG2 cells (the top row), HepG2 cells pretreated with LBA for 4 h (the middle row), and MCF-7 cells (the bottom row) cultured with CeONR@PDA-Gal/Hyp for 4 h. (**b**) Quantitative analysis of the fluorescence of Hyp in different types of the cells by Image-Pro Plus (n = 50).

The surface of HepG2 cells contains overexpressed asialo-glycoprotein receptors (ASGP-R), which can be specifically recognized by D-(+)-Galactose [38]. Therefore, Gal-SH, a derivative of D-(+)-Galactose, was synthesized and used to modify the surface of nanorods to endow their ability to target HepG2 cells. To evaluate the targetability of CeONR@PDA-Gal/Hyp, HepG2 cells and MCF-7 cells were co-cultured with CeONR@PDA-Gal/Hyp for 4 h, and the target effect of CeONR@PDA-Gal/Hyp was evaluated by CLSM. As shown in Figure 4a, the fluorescence of Hyp in HepG2 cells was relatively stronger than that in MCF-7 under the same conditions. This is due to MCF-7 cells have relatively lower ASGP-R expression on their membrane surface in comparison with HepG2 [39]. To further prove the function of D-(+)-Galactose in facilitating the cellular uptake by specifically recognizing ASGP-R receptors, HepG2 cells were pretreated with lactobionic acid (LBA) for 4 h. The fluorescence of Hyp observed, thus, was clearly weaker when compared with that in non-pretreated HepG2 cells, and similar to that in MCF-7. The pathway of CeONR@PDA-Gal/Hyp entering HepG2 cells mainly relied on the active targeted transport of ASGP-R regulation, which enhanced the cellular uptake of CeONR@PDA-Gal/Hyp. The galactose receptor of HepG2 can be blocked by pretreating the cells with LBA, which inhibits the active targeted transport pathway. CeONR@PDA-Gal/Hyp was taken up by HepG2 cells pretreated with LBA and MCF-7 cells mainly via non-specific endocytosis. Therefore, intracellular fluorescence was relatively weak. Meanwhile, the CLSM data were semi-quantitatively analyzed using Image-Pro Plus software (Figure 4b), where a similar profile of fluorescence intensity was obtained. 

### 3.4. Intracellular ROS Production

**Figure 5:**
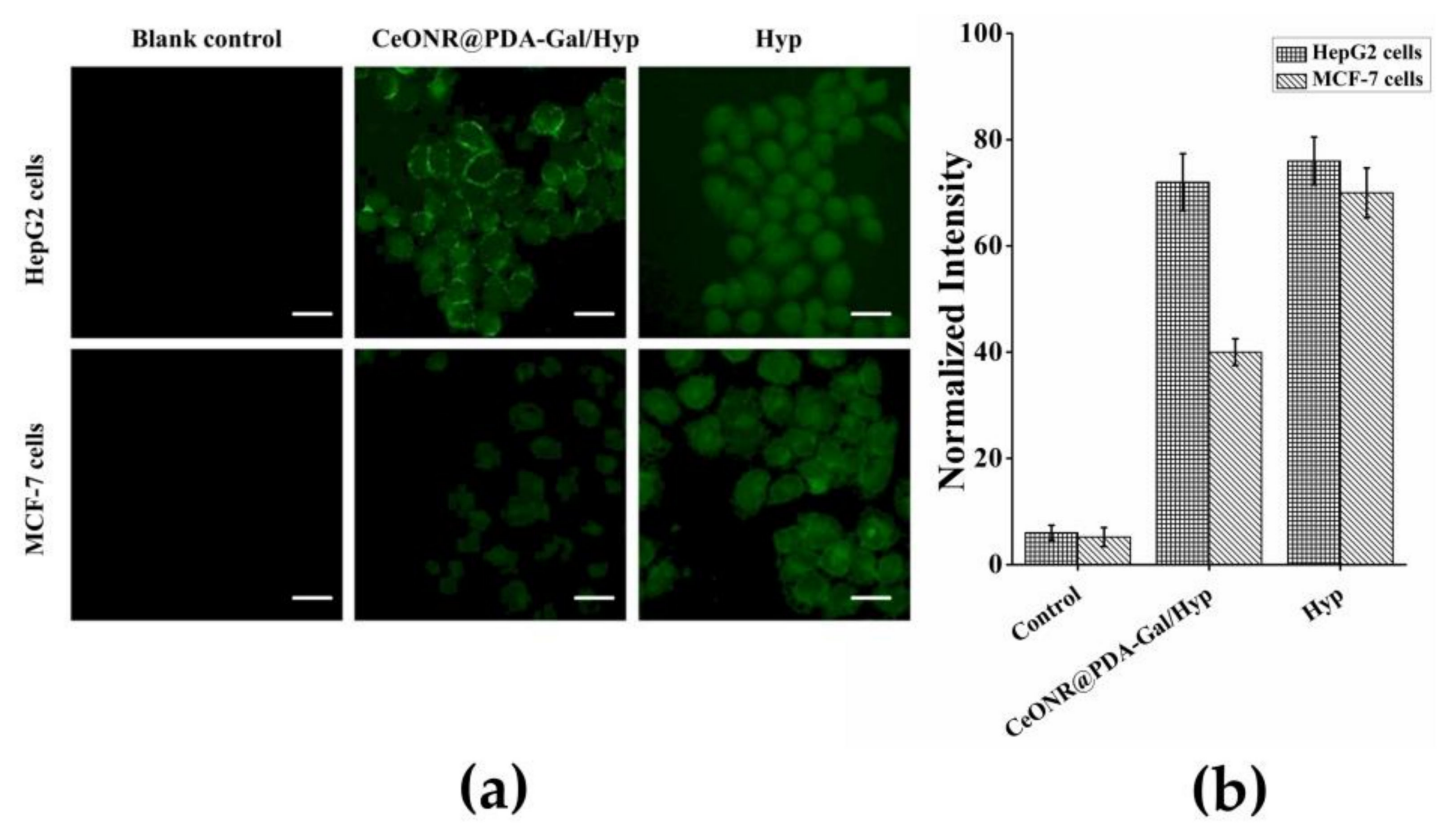
(**a**) The fluorescence intensity of DCF in HepG2 cells and MCF-7 cells was observed by fluorescence microscopy. Scale bar: 20 μm. (**b**) Quantitative analysis of the fluorescence intensity of DCF in HepG2 cells and MCF-7 cells by Image-Pro Plus (n = 50).

It has been reported that Hyp can induce apoptosis under light conditions, mainly due to the activation of the mitochondrial apoptotic pathway by ROS produced by a photoreaction [40]. Therefore, for PDT, the amount of ROS in the cells is one of the important factors for examining the photodynamic effect. DCFH-DA was used as an ROS fluorescent indicator to examine the amount of intracellular ROS. DCFH-DA does not fluoresce in the cell, but it can be hydrolyzed by intracellular esterase to form DCFH, which can then be oxidized by ROS to form DCF, which emits green fluorescence upon excitation by a 488-nm wavelength. Therefore, the fluorescence intensity of DCF is directly proportional to the amount of ROS in the cells. The results were shown in Figure 5a, where it could be seen that the green fluorescence in the PBS-treated control group was neglected, while the fluorescence in HepG2 cells and MCF-7 cells treated with Hyp and irradiated with a 590-nm light source for 30 min was strong with a similar intensity. However, under the same conditions, when CeONR@PDA-Gal/Hyp was co-cultured with HepG2 cells and MCF-7 cells for 4 h, the fluorescence intensity in HepG2 cells was significantly stronger than that in MCF-7 cells. The results not only proved that CeONR@PDA-Gal/Hyp could enter HepG2 cells and achieve an ideal photodynamic effect under irradiation, but also further demonstrated the targeting ability of CeONR@PDA-Gal/Hyp to HepG2 cells. Further, the fluorescence microscopy data were semi-quantitatively analyzed using Image-Pro Plus software (Figure 5b), which clearly showed CeONR@PDA-Gal/Hyp could not only produce a larger amount of ROS in HepG2 cells than in MCF-7 cells, but also the ROS yield was almost the same as that of free Hyp.

### 3.5. Phototoxic, Dark Toxic, and Material Toxic Effects

**Figure 6:**
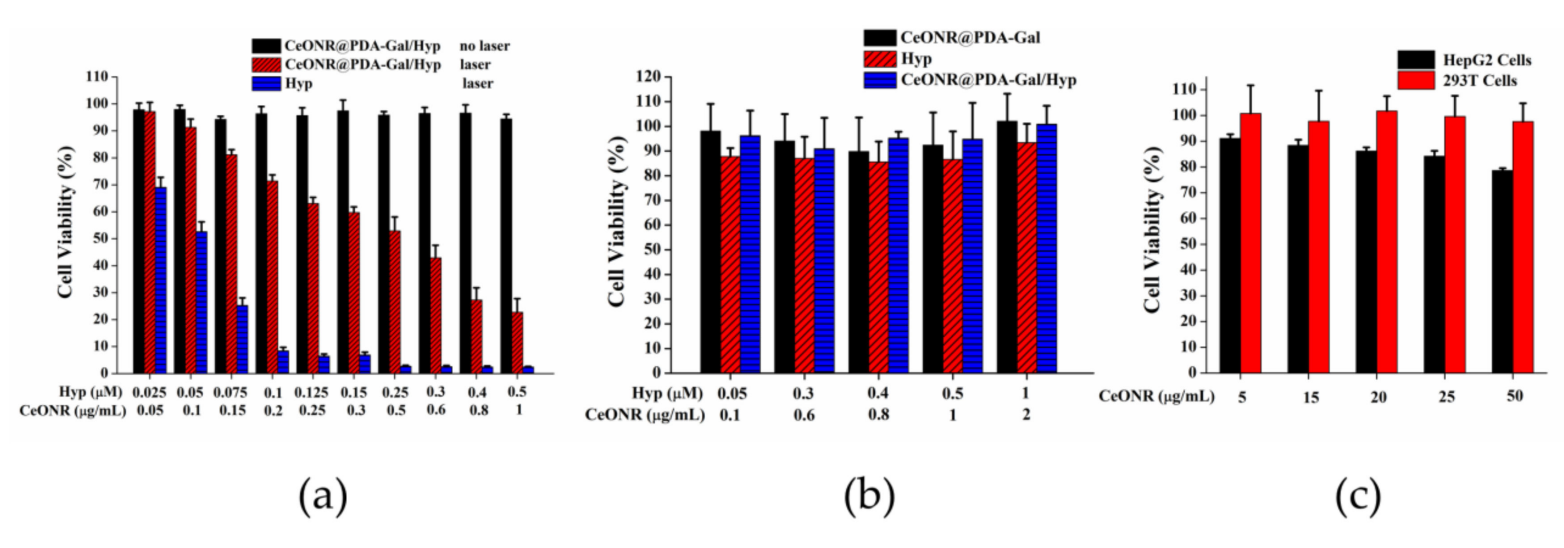
(**a**) The cell viability of HepG2 cells, which were co-cultured with Hyp and CeONR@PDA-Gal/Hyp (Hyp 0.025–0.5 μM) for 4 h, respectively, irradiated with a 590-nm light source (8.6 mW/cm^2^) for 30 min, and then cultured for 24 h in the dark. HepG2 cells co-cultured with CeONR@PDA-Gal/Hyp at the same concentrations for 28 h in the dark were used as control. (**b**) The cell viability of 293T cells treated with different concentrations of free Hyp, CeONR@PDA-Gal, and CeONR@PDA-Gal/Hyp, respectively, without laser exposure for 24 h. (**c**) The cell viability of HepG2 cells and 293T cells treated with CeONR@PDA-Gal at various doses (CeONR 5–50 μg/mL) without laser irradiation.

To study the phototoxic, dark toxic, and material toxic effects, HepG2 cells were incubated with CeONR@PDA-Gal/Hyp and Hyp, respectively, for 4 h and irradiated with a 590-nm light source for 30 min. Phototoxicity was then examined by MTT assay (Figure 6a). It could be seen that the cell viability of HepG2 was higher than 94% in all the concentrations studied (relate Hyp from 0.025 to 0.5 μM) under a dark condition, but decreased quickly with the increase of the concentration of Hyp under irradiation. The IC_50_ of CeONR@PDA-Gal/Hyp was determined to be 0.278 μM (Figure S3). While at a similar concentration of hematoporphyrin (Hp, 0.25μM), a commercial photosensitizer, the cell viability of HepG2 cells was as high as 94% (Figure S4). This indicates that so-prepared CeONR@PDA-Gal/Hyp has excellent photodynamic therapy efficiency, even though it is not comparable with that of free Hyp. The reason for this might result from the real concentration of Hyp in the cells treated with CeONP@PDA-Gal/Hyp was different from that with free Hyp at the moment of the measurement. Free Hyp can be taken up directly by the cells, but CeONR@PDA-Gal/Hyp needs time to allow Hyp to be released, which means the real concentration of Hyp in the latter case is much lower than the former, considering its very slow release profiles (see Figure S2). Actually, the increase of the fluorescence of Hyp with time (Figure 3) clearly proved this speculation.

On the other hand, the investigation of the cell viability on 293T cells under the same conditions showed that CeONR@PDA-Gal/Hyp caused much lower cytotoxicity in comparison with free Hyp (Figure S5), which implies CeONR@PDA-Gal/Hyp can reduce side effects of Hyp to normal cells. 

The dark cytotoxicity of CeONR@PDA-Gal and CeONR@PDA-Gal/Hyp, at different concentrations, were studied and compared with Hyp by using 293T cells. The experimental results revealed that, not like Hyp, which showed a slight dark cytotoxicity (the cell viability was reduced by about 10% corresponding to the varied concentration of Hyp), CeONR@PDA-Gal and CeONR@PDA-Gal/Hyp showed neglectable dark cytotoxicity to 293T cells (Figure 6b). Similar results were obtained when 0.25 μM CeONR@PDA-Gal/Hyp and Hyp were co-cultured with 293T cells for 24, 48, and 72 h without laser irradiation (Figure S6). This further confirmed that CeONR@PDA-Gal is an ideal Hyp transport carrier.

In the acidic microenvironment of the tumor, the oxidation activity of CeONR can be stimulated, which induces tumor cells to produce ROS, and then promote apoptosis. Therefore, CeONR could exhibit chemical toxicity to tumor cells. To find out if this is the case with our system, CeONR@PDA-Gal (CeONR 5–50 μg/mL) was incubated with HepG2 cells and 293T cells, respectively. The results were shown in Figure 6c, where the cell viability of HepG2 cells was reduced by 10%–20% at the studied range of concentration, while that of 293T cells was kept intact. This result coincided with what was reported previously [23,24,25,26] and further proved that CeONR@PDA-Gal could be used not only as a carrier for photosensitizers, but also as a chemotherapeutic factor.

### 3.6. Cell Apoptosis

**Figure 7:**
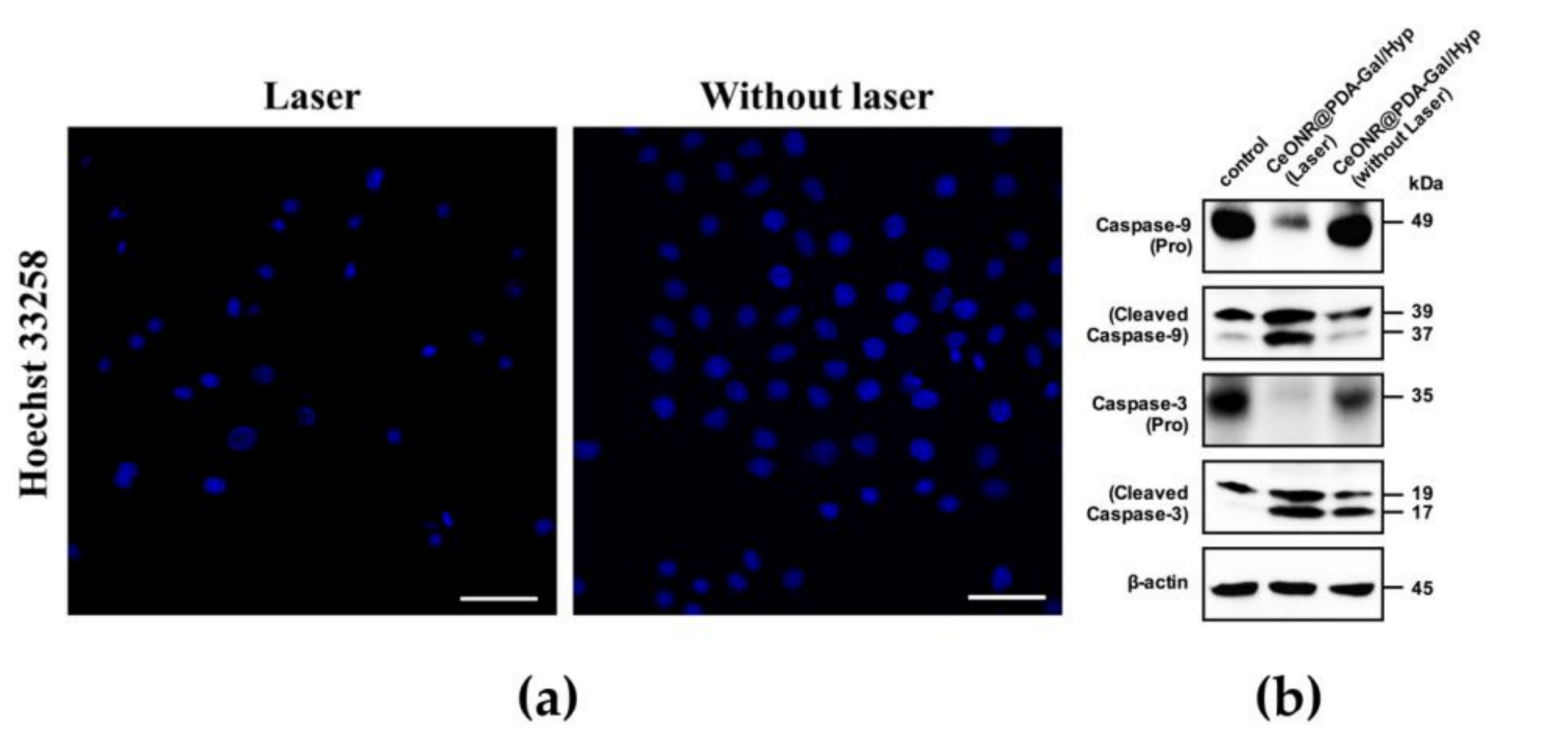
(**a**) CLSM images of the nuclei of HepG2 cells cultured with CeONR@PDA-Gal/Hyp (Hyp 0.25 μM) with or without laser irradiation. Scale bar: 50 μm. (**b**) Western blot assay for Caspase-3 and Caspase-9 in HepG2 cells treated with CeONR@PDA-Gal/Hyp (Hyp 0.5 μM) for 4 h, and with or without laser irradiation, then cultured for 24 h (PBS served as a blank control, β-actin served as an inner control).

The apoptosis of HepG2 cells after photodynamic therapy was examined by observing changes of nuclear morphology as an indicator of apoptosis. The nucleus morphology of cells in the stage of apoptosis or necrosis, such as nuclear pyknosis, nuclear rupture, and nuclear lysis, is significantly different from that of the normal nucleus. As shown in Figure 7a, when HepG2 cells were cultured with CeONR@PDA-Gal/Hyp and then exposed to irradiation, the morphology of the nuclei showed significant nuclear pyknosis, which revealed the apoptosis of HepG2 cells. However, the morphology of the nuclei without laser irradiation was intact. The results were consistent with the MTT experiments.

To further examine the effect of drug-induced apoptosis of tumor cells, levels of the two key ingredients of the apoptotic pathway were analyzed by using Western blot. As shown in Figure 7b, Caspase-9, as an essential initiator caspase of the intrinsic apoptotic pathway, was activated after light treatment. Caspase-3, as a downstream target of activated Caspase-9, was also activated by photo induction. These data confirmed that light induced a significant increase of apoptosis compared to the controls. 

**Figure 8:**
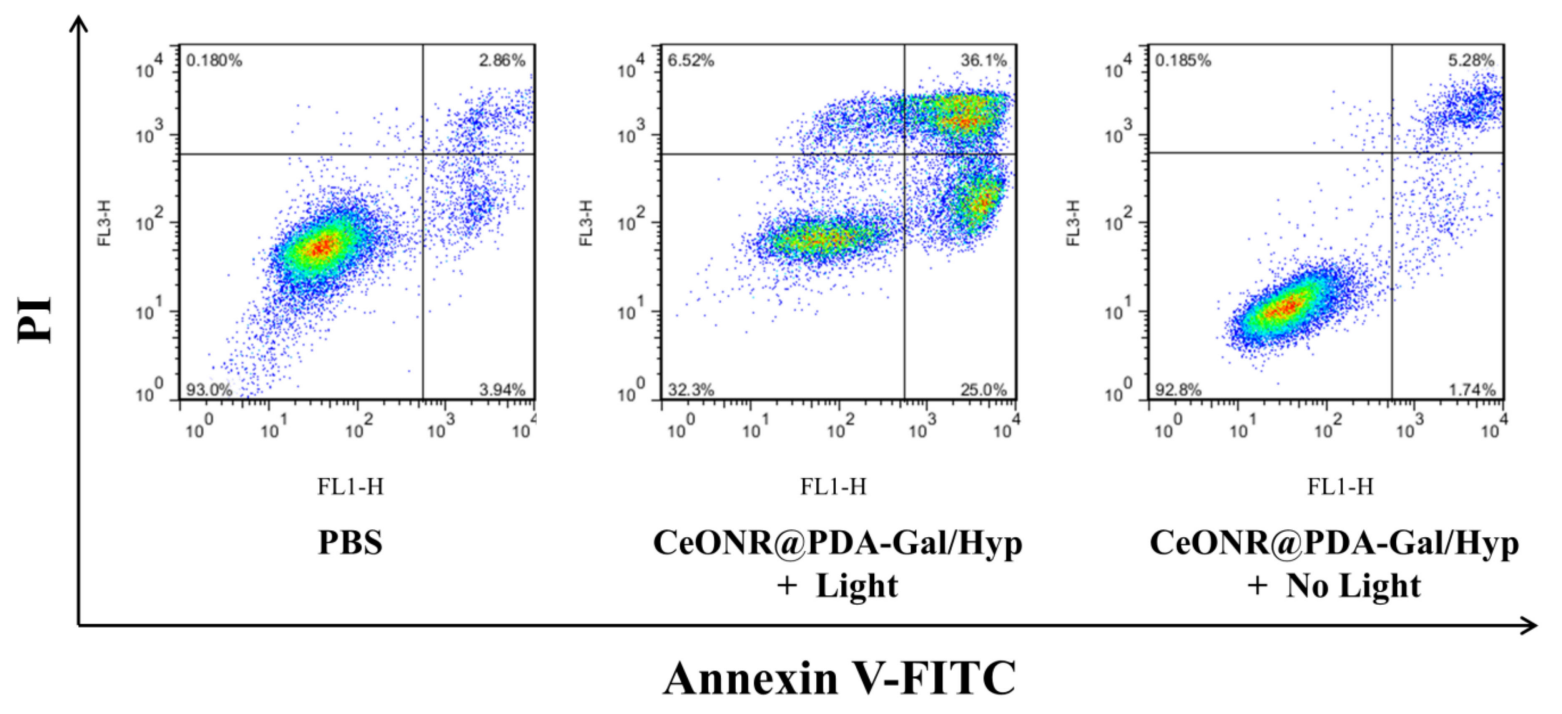
The cell death and apoptosis of HepG2 cells after staining with annexin V–FITC and propidium iodide (PI) by flow cytometry.

Additionally, the photodynamic therapy effect of CeONR@PDA-Gal/Hyp on HepG2 cells was investigated with Annexin V conjugate staining assay. As shown in Figure 8, the cell death when treated without laser was 6.70%, whereas, with laser irradiation, the cell death increased to 61.10%. Taken together, it appeared that CeONR@PDA-Gal/Hyp could effectively induce apoptosis of HepG2 cells with laser irradiation, while the toxicity to HepG2 cells was weak without irridiation.

## 4. Conclusions

In summary, a novel Hyp delivery system of CeONR@PDA-Gal/Hyp based on a thiolated galactose modified PDA-coated CeONR has been constructed. CeONR@PDA-Gal/Hyp not only showed targeting ability to ASGP-R overexpressing HepG2 cells, but also mitigated the self-aggregation of the photosensitizer under physiological conditions. The fluorescence was quenched with negligible dark toxicity when Hyp was loaded on the CeONR@PDA-Gal. Hyp could be released after the nanorods entered tumor cells to produce abundant ROS under 590-nm light irradiation and led to high tumor cells’ killing ability. CeONR@PDA-Gal can be utilized as a targeted photosensitizer delivery system with a potential for a synergistic therapeutic effect. Hence, this work exemplifies a novel targeted delivery of hydrophobic photosensitizers for cancer treatment based on a scaffold that acts as more than a carrier.

## Figures and Tables

**Table 1:** The Zeta potential of nanorods.

Nanorods	Zeta Potential (mV)
CeONR	−8.46 ± 0.7
CeONR@PDA	−12.55 ± 1.2
CeONR@PDA-Gal	−16.33 ± 0.9
CeONR@PDA-Gal/Hyp	−32.59 ± 0.5

## Supplementary Materials

The following are available online at https://www.mdpi.com/2073-4360/11/6/1025/s1. Scheme S1: Synthesis of Compound Hyp (3). Scheme S2: Synthesis of Compound Gal-SH. Figure S1: FT-IR spectra of CeONR, CeONR@PDA, CeONR@PDA-Gal and CeONR@PDA-Gal/Hyp, Figure S2: Release profiles of Hyp from CeONR@PDA-Gal/Hyp in varied pH. Figure S3: The median lethal dose of HepG2 cells treated with CeONR@PDA-Gal/Hyp under 590 nm light source (8.6 mW/cm^2^) for 30 min. Figure S4: Hp and HepG2 cells were co-cultured for 4 h, and then irradiated with a 630 nm light source (8.6 mW/cm^2^) for 30 min, and the cell viability after 24 h of culture. Figure S5: Cell viability of free Hyp and CeONP@PDA-Gal/Hyp at different concentrations to HepG2 cells with/without irradiation of 590 nm. Figure S6: 293T cells treated with free Hyp and CeONR@PDA-Gal/Hyp (Hyp 0.25 μM) without laser exposure for 24, 48, and 72 h.
