# Supplementary material for: A Hypericin Delivery System Based on Polydopamine Coated Cerium Oxide Nanorods for Targeted Photodynamic Therapy
Source: Polymers (Basel). 2019 Jun 10;11(6):1025. doi: 10.3390/polym11061025 (PMC6630464; doi:10.3390/polym11061025)
Supplement: Supplementary file 1 [file polymers-11-01025-s001.pdf]

polymers

MDPI

Supporting Materials: A Hypericin Delivery System Based on Polydopamine Coated Cerium Oxide Nanorods for Targeted Photodynamic Therapy

Yang Wang †, Yu Zhang †, Ming Jin, Yinghua Lv, Zhichao Pei and Yuxin Pei *

Shannxi Key Laboratory of Natural Products & Chemical Biology, College of Chemistry & Pharmacy, Northwest A&F University, Yangling 712100, China;
peiyx@nwafu.edu.cn; Tel.: +86-29-8709-1196; Fax: +86-29-8709-2769

Synthesis and characterization of the compounds

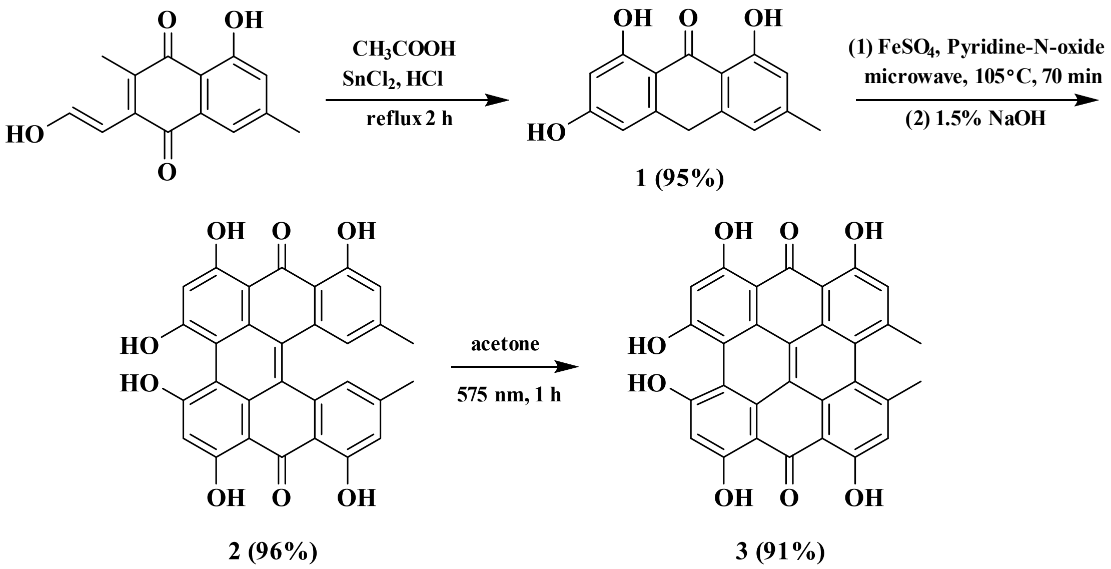

Scheme S1. Synthesis of Compound Hyp (**3**).

Emodine (0.68 g, 2.5 mmol) and ethanoic acid (50 mL) were added into the round bottom flask to heat up and reflow under stirring conditions. $SnCl_2 \cdot 2H_2O$ (3.95 g, 17.5 mmol) was added to concentrated hydrochloric acid (25 mL) and stirred to dissolve it. Then the mixture was slowly dropped to the round bottom flask, continue to reflux reaction for 2 h. At the end of the reaction the heating was stopped and the reaction system was cooled to room temperature and poured into ice water (150 mL). A large amount of yellow precipitation was observed. The filter cake was washed to neutral with water, and the yellow coarse product was dried overnight in the vacuum drying oven. Emodin anthrone **1** was obtained by column chromatography (0.61 g, 95%) [1]. ^{1}H NMR (500 MHz,

DMSO-d6) δ 12.37 (s, 1H), 12.21 (s, 1H), 10.83 (s, 1H), 6.77 (s, 1H), 6.67 (s, 1H), 6.41 (s, 1H), 6.22 (s, 1H), 4.29 (s, 2H), 2.31 (s, 3H) ppm.

In 10 mL of quartz tube, emodin anthrone **1** (0.26 g, 1 mmol), ferrous sulfate (0.022 g, 0.08 mmol), sodium hydroxide (60 mg, 1.5 mmol) and pyridine nitrogen oxides (0.50 g, 5.26 mmol) were dissolved in distilled water (4 mL). The reaction system was placed in a microwave reactor at 10 W, 105 °C under argon atmosphere for 70 min. At the end of the reaction, the reaction system was acidified with dilute hydrochloric acid (3%), then settled and precipitated. The solid was collected by filtration, and the filter cake was washed with water to be neutral and vacuum dried. The purple protohypericin **2** (0.24 g, 96%) was obtained by column chromatography[1]. ^{1}H NMR (500 MHz, DMSO-d$_6$) δ 14.47 (s, 2H), 12.99 (s, 2H), 7.20 (s, 2H), 6.74(s, 2H), 6.32 (s, 2H), 2.05 (s, 6H) ppm.

In a round bottom flask, protohypericin **2** (0.24 g, 0.48 mmol) was dissolved into acetone (200 mL). The reaction system was irradiated with a 575 nm light source for 1 h in an argon atmosphere. The hypericin **3** (0.22 g, 91%) was obtained by column chromatography [1]. ^{1}H NMR (500 MHz, DMSO-d$_6$) δ 14.72 (s, 2H), 14.08 (s, 2H), 7.42 (s, 2H), 6.56 (s, 2H), 2.73 (s, 6H) ppm.

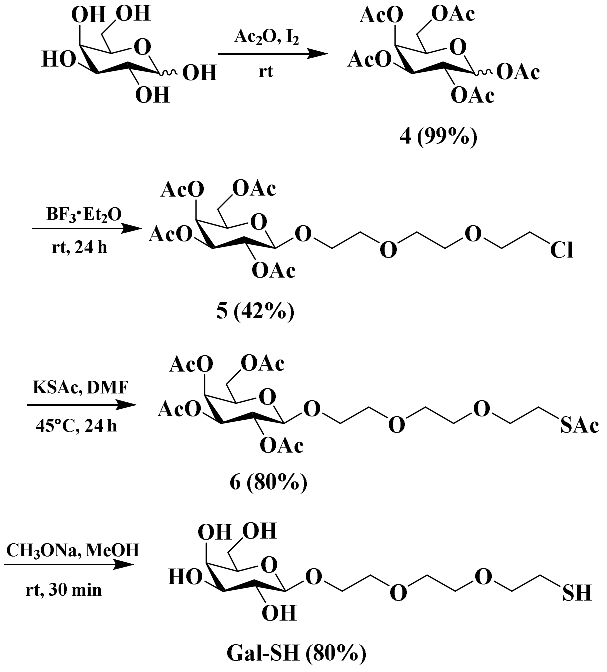

Scheme S2. Synthesis of Compound **Gal-SH.**

D-galactose (5.00 g, 27.8 mmol), I_2 (0.11 g, 0.44 mmol) and acetic anhydride (50 mL, 0.5 mol) were added into 100 mL round bottom flask. The mixed system reacts at room temperature until all the solids are completely dissolved. It was then extracted three times with CH_2Cl_2 and the CH_2Cl_2 organic phase was collected then washed with saturated Na_2SO_3 aqueous solution and saturated $NaHCO_3$ aqueous solution, respectively. After that,

the organic phase was dried over anhydrous Na_2SO_4 and concentrated under reduced pressure to give Compound **4** (10.72 g). Then, peracetylated galactose (5.00 g) and 2-chloroethoxy-2-ethoxydiethanol (2.75 mL, 19.13 mmol) were dissolved with dry dichloromethane (30 mL) in a 50 mL round bottom flask. Furthermore, $BF_3 \cdot Et_2O$ (8 mL, 64 mmol) was added dropwise under ice-cooling. Then, the reaction system was stirred at room temperature for 24 h. At the end of the reaction, additional dichloromethane (30 mL) was added to the system and washed three times with saturated $NaHCO_3$. The organic phase was collected and dried over anhydrous Na_2SO_4. Concentrate to get the crude product. Separation by column chromatography gave Compound **5** (3.15 g, 42%) [2]. ^1H NMR (500 MHz, $CDCl_3$) δ 5.46 (d, J = 3.2 Hz, 1H), 5.37 (dd, J = 10.8, 3.3 Hz, 1H), 5.17 (d, J = 3.6 Hz, 1H), 5.13 (dd, J = 10.8, 3.6 Hz, 1H), 4.30 (t, J = 6.6 Hz, 1H), 4.14-4.06 (m, 3H), 3.81-3.61 (m, 9H), 2.14 (s, 3H), 2.08 (s, 3H), 2.05 (s, 3H), 1.99 (s, 3H) ppm.

In a 100 mL round bottom flask, compound **5** (3.00 g, 6 mmol) and potassium thioacetate (2.05 g, 18 mmol) were dissolved in dry DMF (45 mL). The reaction was stirred at 45 °C for 24 h. The reaction system was diluted with ethyl acetate, washed twice with water, and washed once with saturated $NaHCO_3$ and saturated brine, respectively. The organic phase was collected and the organic phase was dried over anhydrous Na_2SO_4 and concentrated to give the crude product. Purification by column chromatography gave Compound **6** (2.60 g, 80%) [2]. ^1H NMR (500 MHz, $CDCl_3$) δ 5.46 (dd, J = 3.4, 1.1 Hz, 1H), 5.37 (dd, J = 10.8, 3.4 Hz, 1H), 5.17 (d, J = 3.7 Hz, 1H), 5.13 (dd, J = 10.8, 3.7 Hz, 1H), 4.30 (m, 1H), 4.15-4.06 (m, 2H), 3.82-3.78 (m, 1H), 3.69-3.66 (m, 3H), 3.65-3.59 (m, 6H), 3.10 (t, J = 6.5 Hz, 2H), 2.34 (s, 3H), 2.14 (s, 3H), 2.08 (s, 3H), 2.04 (s, 3H), 1.98 (s, 3H) ppm..

In a 25 mL round bottom flask, Compound **6** (0.40 g, 0.74 mmol) and CH_3ONa (0.060 g, 1.1 mmol) were dissolved in methanol (10 mL). The reaction was stirred at room temperature for 30 min. The cation exchange resin was then added and stirred until the solution was neutral. Filter to remove cation exchange resin. The organic phase was collected and concentrated to give the crude product. Purification by column chromatography gave Gal-SH [2]. ^1H NMR (500 MHz, D_2O) δ 5.02 (d, J = 3.8 Hz, 1H), 4.04-3.91 (m, 4H), 3.9-3.69 (m, 4H), 3.01 (t, J = 6.0 Hz, 1.5H), 2.87 (t, J = 6.3 Hz, 0.5H) ppm.

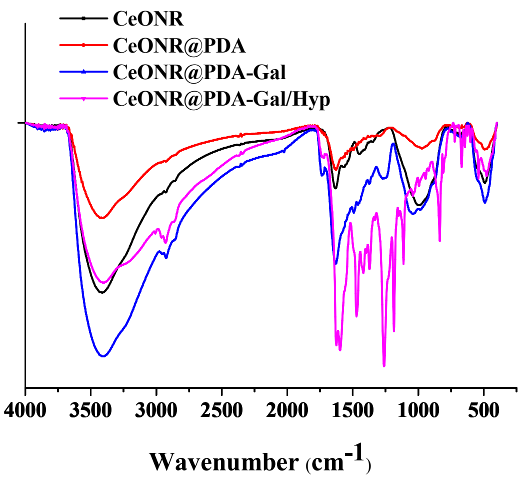

Figure S1. FT-IR spectra of CeONR, CeONR@PDA, CeONR@PDA-Gal and CeONR@PDA-Gal/Hyp.

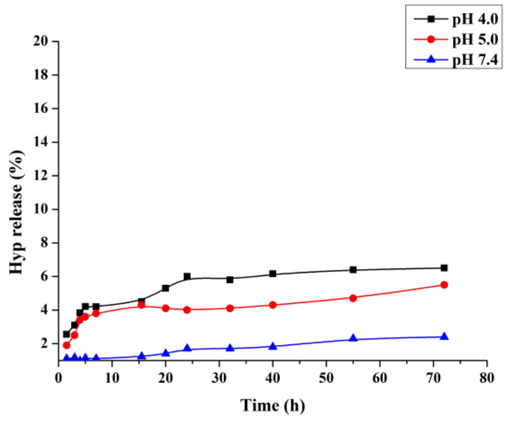

Figure S2. Release profiles of Hyp from CeONR@PDA-Gal/Hyp in varied pH.

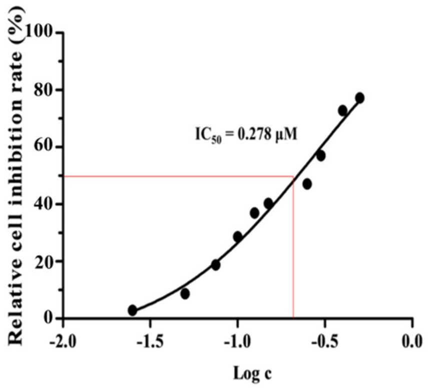

Figure S3. The median lethal dose of HepG2 cells treated with CeONR@PDA-Gal/Hyp under 590 nm light source (8.6 mW/cm^2) for 30 minutes.

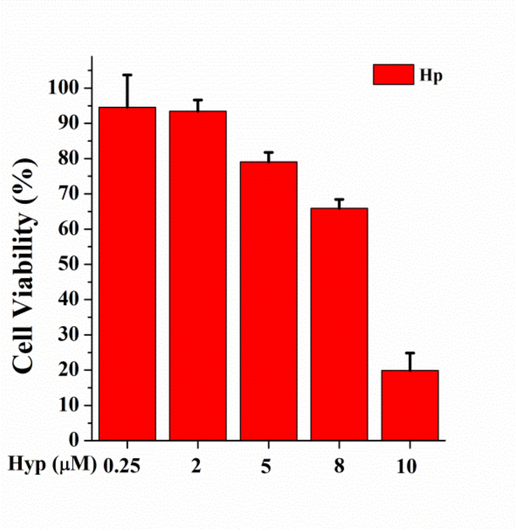

Figure S4. Hp and HepG2 cells were co-cultured for 4 h, and then irradiated with a 630 nm light source (8.6 mW/cm^2) for 30 min, and the cell viability after 24 h of culture.

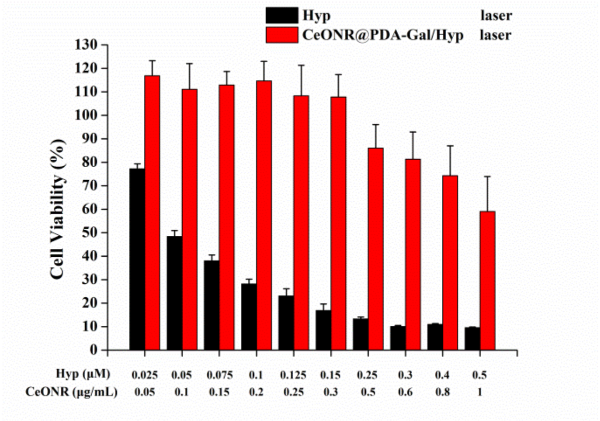

Figure S5. Cell viability of free Hyp and CeONP@PDA-Gal/Hyp to 293T with irradiation of 590 nm.

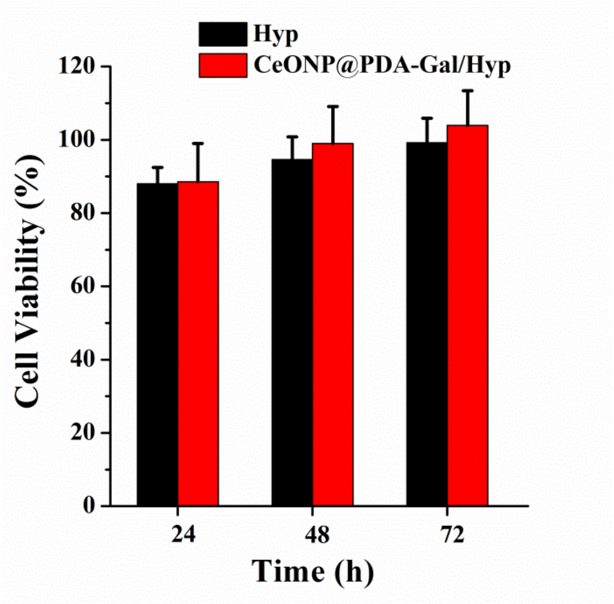

Figure S6. 293T cells treated with free Hyp and CeONR@PDA-Gal/Hyp (Hyp 0.25 μM) without laser exposure for 24, 48 and 72 h.

References:

1. Y. Zhang, K. Shang, X. Wu, S. Song, Z. Li, Z. Pei and Y. Pei, *Rsc Advances*, 2018, **8**, 21786-21792.
2. B. L. Ferla, G. D'Orazio, G. Zotti, and B. Vercelli, *Electroanalysis,* 2018, **30**, 798 –802.
